# Comparison of two point-of-care lung ultrasound techniques and their associated outcomes for bronchiolitis in the pediatric emergency department

**DOI:** 10.1186/s13089-025-00410-y

**Published:** 2025-01-17

**Authors:** Jaron A. Smith, Michael C. Cooper, Kenneth Yen, Joan Reisch, Bethsabee S. Stone

**Affiliations:** 1 Department of Emergency Medicine, Phoenix Children’s Hospital, 1919 E Thomas Rd, Phoenix, AZ 85016 USA; 2 Baystate Medical Center, Department of Emergency Medicine, Division of Pediatric Emergency Medicine, Springfield, MA USA; 3 Department of Pediatrics, Division of Emergency Medicine, University of Texas Southwestern, Children’s Medical Center, Dallas, TX USA; 4 School of Public Health, Division of Statistics, University of Texas Southwestern, Dallas, TX USA

**Keywords:** Pediatrics, Emergency medicine, Bronchiolitis, Lung ultrasound, Point-of-care ultrasound

## Abstract

**Background:**

Acute bronchiolitis (AB) is the most common lower respiratory tract infection in infants. Clinician diagnosis and management vary due to limited objective assessment tools. Point-of-care lung ultrasound (LUS) offers a promising diagnostic and prognostic tool in the emergency department (ED), however, the time to perform LUS is of concern in the emergency setting.

**Methods:**

Infants ≤ 12 months diagnosed with AB in the emergency department were enrolled. Two LUS techniques were performed sequentially: a 12-segment “lawnmower” approach and a posterior paravertebral “waterfall” technique. LUS were scored (0–36 for lawnmower; 0–6 for waterfall). Respiratory support (RS) was categorized into three levels: no RS (room air), low RS (wall O2 or heated high flow nasal cannula < 1L/kg), and high RS (heated high flow nasal cannula ≥ 1L/kg or positive pressure). Clinical data, including RS at 12 and 24 h, maximum RS, disposition, and length of stay, were extracted via chart review and compared to mean LUS scores for each technique. Calculated areas under the curve (AUC) were compared using the Youden Index (*J*).

**Results:**

82 infants were enrolled. The mean waterfall scanning time was 1.65 min (SD 0.55) compared to the lawnmower’s 7.65 min (SD 1.45). The difference between mean LUS scores for the waterfall technique was statistically significant for all disposition comparisons and nearly all RS comparisons. While the lawnmower AUC was greater than the waterfall AUC for all RS and disposition comparisons, the Youden Index (*J)* was statistically significantly different for only two of the eight comparisons.

**Conclusion:**

The posterior-only LUS technique is faster than the lawnmower technique, provides comparable information for disposition, and has a stronger association with LOS, but is less associated with RS. The waterfall technique may be a suitable alternative to more time-intensive, thorough techniques.

**Supplementary Information:**

The online version contains supplementary material available at 10.1186/s13089-025-00410-y.

## Introduction

Acute bronchiolitis (AB) is a well-described viral lower respiratory infection characterized by respiratory distress symptoms, including tachypnea, retractions, and wheezing [1–3]. It is the most common lower respiratory tract infection in infants, rarely affecting children older than two to three years [3, 4].

AB is diagnosed clinically, but its management varies due to the absence of standardized clinical assessment tools and the limited role of radiography in diagnosis [2, 5, 6]. The use of point-of-care lung ultrasound (LUS) in the emergency department (ED) is promising because of its safety profile and dynamic bedside acquisition to make immediate decisions [7–11]. Recently, the authors of this study described the favorable role of bedside LUS in diagnosing and predicting outcomes in AB, specifically using a scoring tool to stratify outcomes of interest to ED clinicians: respiratory support (RS) at 12 and 24 h, maximum RS, ED disposition, and hospital length of stay (LOS) [12]. Other studies have also recently emerged with similar findings supporting the use of LUS in AB [7, 11, 13–21].

There is no standard technique for LUS acquisition in AB, although several have been described [7, 11, 14, 19–23]. Additionally, the time required to perform LUS can be restrictive for the busy ED clinician [24–26]. Some studies suggest that LUS of the posterior lung fields alone is sufficient to predict severity in AB; however, these studies involved only younger infants who are primarily supine [7, 13, 27].

To our knowledge, no studies have directly compared two LUS techniques in AB. Our primary goals were to evaluate associations of RS, disposition, and LOS with a posterior-only LUS technique. Our secondary goals were to compare two techniques with these outcomes and analyze the time required to complete the LUS examination for each.

## Methods

### Study design and setting

This investigation was a planned secondary analysis of data from a prospective, observational study of a convenience sample of pediatric patients presenting to a level one trauma center, tertiary/quaternary ED. Enrollment was from June 12, 2022 to October 11, 2022 [12]. Appropriate Institutional Board Review was obtained.

### Study protocol

#### Selection of participants

Inclusion and exclusion criteria were described previously [12]. In brief, patients aged 12 months and younger diagnosed with AB by their treating ED clinician were enrolled after obtaining written informed consent. All scans were obtained during subjects’ ED stay. Patients were excluded for immunodeficiency/immunosuppression, moderate to severe prematurity (< 34 weeks), chronic pulmonary disease, chronic moderately to severely depressed heart function based on most recent echocardiogram, sickle cell disease, chronic neuromuscular disease, or diagnosis of pneumonia within 14 days prior to ED presentation.

#### LUS techniques

**Fig. 1:**
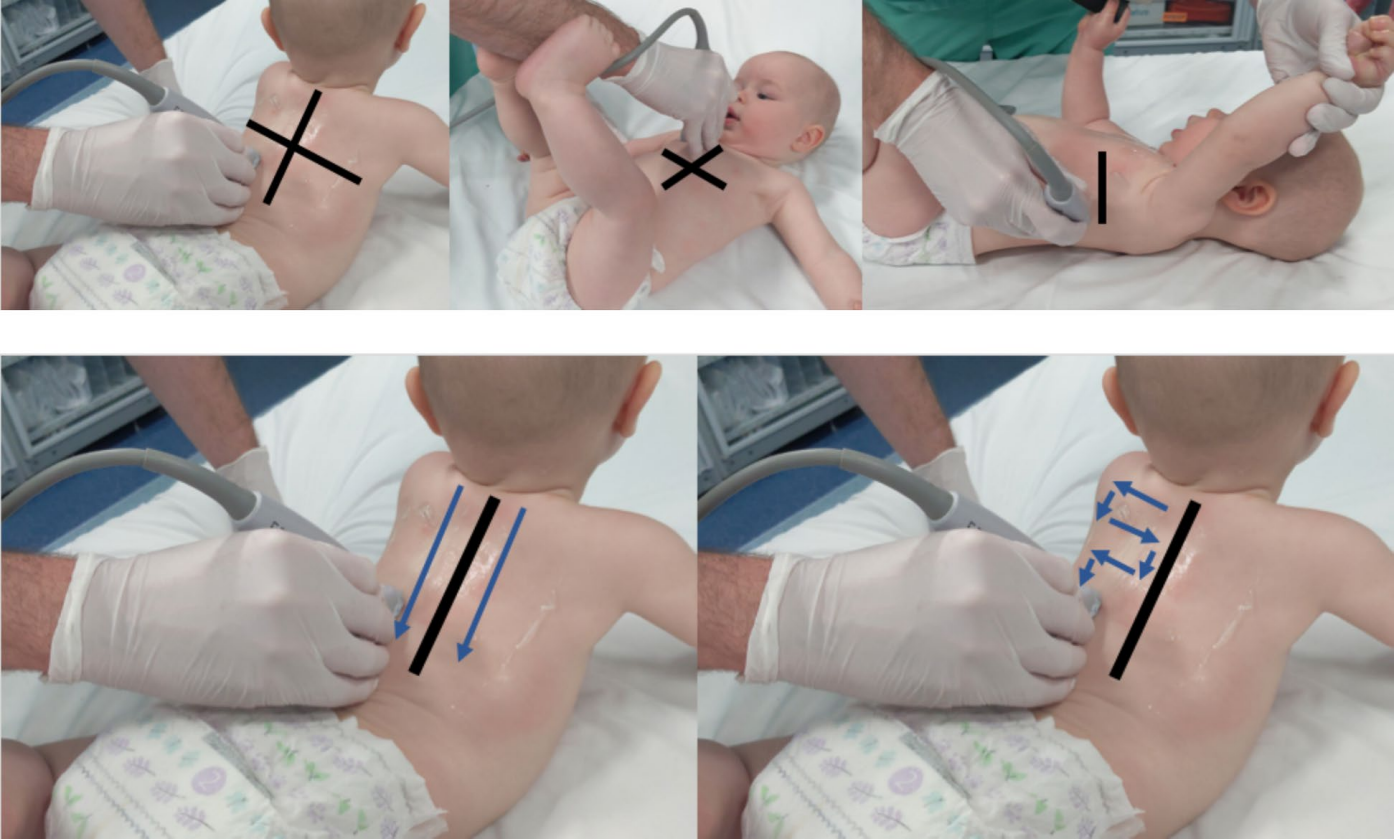
Division of scanning fields of the lawnmower technique (1a), as previously reported.^12^, and side-by-side comparison of transducer movement of the waterfall technique (left) and lawnmower technique (right)

The two LUS techniques were performed sequentially. First, a “lawnmower” approach was used as described in our previous manuscript and other studies [12, 22, 28, 29] where we divided the anterior, lateral, and posterior lung fields superiorly and inferiorly for a total of 12 lung fields (Fig. 1a). Once lawnmower acquisition was complete, a posterior paravertebral “waterfall” technique was performed. The transducer was held also in a longitudinal position at the superior aspect of one of the posterior lung fields, between the medial border of the scapula and the spine. Rather than scanning side-to-side, the transducer instead was slowly dragged directly inferiorly towards the diaphragm in one motion. This process was repeated on the contralateral posterior lung field (Fig. 1b). A similar technique was described by Gori et al. [15]. Previous publications have described the posterior lung fields on LUS as most reflective of illness severity in AB [7, 13].

All LUS videos were obtained during the ED visit by a pediatric emergency medicine fellow who demonstrated lung ultrasound competency prior to the study [30], or one of two pediatric emergency medicine physicians with fellowship training in point-of-care ultrasound. Videos were obtained using a Sonosite X-porte with the L25 × 12–6 Hz linear transducer.

#### LUS scoring and documentation

**Fig. 2:**
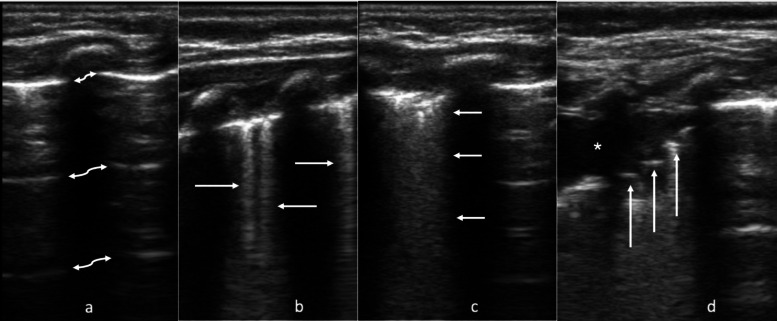
- Four lung ultrasound findings. Example of the four lung ultrasound findings and associated scores, as previously reported^12^. (a) shows A lines (white arrows) with < 3 B lines (no B lines shown in this image), scores 0, (b) shows ≥ 3 B lines per lung segment (3 B lines shown in this image by white arrows), scores 1, (c) shows consolidated B lines, or “white out,” (white arrows) scores 2, (d) shows subpleural consolidation (shown with white *) with consolidated B lines, scores 3. In this final image, white arrows indicate air bronchograms, a common feature in subpleural consolidations

The scoring for each lung field [11, 12, 29, 31] is shown in Fig. 2 and described below:

Score of 0: normal lung sliding, mostly A lines, and/or < 3 B lines per lung segment.

Score of 1: ≥ 3 B lines per lung segment, but not consolidated/ “white out”.

Score of 2: consolidated B lines/“white out,” but no subpleural consolidation or pleural effusion.

Score of 3: subpleural consolidation with any of the findings of score 1 or 2.

The total score range for the two lung fields for the waterfall technique was 0–6, and for the 12 lung fields for the lawnmower technique was 0–36. The scores were summated. Start and end times for both techniques were documented, rounded to the nearest minute, using the machine’s captured video clip times (Supplement 1).

#### Inter-rater reliability and quality control

Within 30 min of completion of the LUS by the primary scanner, a second physician sonographer repeated the scan on a random convenience sample of subjects. Additionally, after patient discharge, all LUS videos were reviewed by a third physician sonographer, blinded to the original scores and patient outcomes. Inter-rater reliability (IRR) comparing the two scanners’ LUS scores, as well as comparing the post-hoc reviewer’s scoring with the scanner’s scoring, was calculated with a linearly weighted Cohen’s kappa. Rating is as follows: ± 0.81 to ± 1.00 excellent, ± 0.61 to ± 0.80 good, ± 0.41 to ± 0.60 moderate.

#### Chart review

The electronic health record was accessed for clinical data ≥ 7 days from hospital discharge. Data was manually extracted and recorded in a secure spreadsheet. These data included: patient demographics; clinical characteristics; ED findings, namely chest X-ray and respiratory viral testing results; ED disposition; and admission and discharge diagnoses. For those admitted, RS at 12 and 24 h, maximum RS during admission, and hospital LOS (measured from time of ED admission order) were recorded.

### Outcome measures

We chose the primary outcome measure as the association of the waterfall LUS score with RS at 24 h, as this seemed the most relevant to the ED clinician, but other pediatric clinicians as well. The secondary outcome was the time required to perform the waterfall and lawnmower techniques. The remainder of comparisons were exploratory and of additional interest to pediatric clinicians. These included RS at 12 h, maximum RS during hospitalization, disposition, and LOS. These are reported together with the comparison of LUS scores with RS at 24 h. Additionally, we compared these findings to the previously described lawnmower technique.

### Data analysis

SAS software version 9.4 (Statistical Analysis System, Cary, NC) and SPSS statistics for Windows, version 29.0.2 (IBM Corp, Armonk, NY) were used for statistical analysis. The Kolmogorov–Smirnov test was used for the normality of data distribution. Values were expressed as means ± standard deviation (SD) for continuous variables, median and interquartile range (IQR) for nonparametric data, or number and percentage (%) for categorical variables. For means, Student's t-test or Mann Whitney U test for two group comparisons and one-way analysis of variance (ANOVA) for more than two groups were used. For categorical variable comparison, chi square contingency table analysis was used. Statistical significance (*p*) was chosen to be 0.05 for both Pearson (normal data) or Spearman (nonparametric data) correlation coefficients.

Initial subdivisions of RS were based on institutional relevance, and included room air (RA), wall oxygen, heated high flow nasal cannula (HHFNC) < 1L/kg, HHFNC 1–2 L/kg, HHFNC > 2L/kg, non-invasive positive pressure, and invasive positive pressure (Supplement 2). Extracorporeal membrane oxygen (ECMO) and death were also considered but none captured in our data set. These subdivisions were combined into three categories for clinical relevance and generalizability: no RS (RA), low RS (wall oxygen or HHFNC < 1L/kg), and high RS (HHFNC ≥ 1L/kg, non-invasive, or invasive positive pressure).

To overcome the challenge of comparing two techniques with different scoring scales (0–6 vs. 0–36), we calculated receiver operating characteristic (ROC) curves with areas under the curve (AUC), and then compared the AUCs using the Youden Index (*J*). This statistical tool is a function of sensitivity and specificity, and often used to measure or summarize diagnostic effectiveness, with values ranging from 0 (limited effectiveness) to 1 (very effective) [32]. Used in our analysis specifically, it allowed for comparison of the ROC curves of the two differing scoring scales. ROC curves could only be generated using binary comparisons, yet there were three levels of RS. We used integer divisions of each possible score for each technique, then compared no RS to any RS, and low RS to high RS, at 12 h, 24 h, and maximum RS, for a total of six ROC curves for RS. This was similarly done with disposition, comparing discharge and admission, as well as floor vs ICU, for a total of two ROC curves for disposition.

## Results

Eighty-two patients were enrolled. Demographic information and clinical characteristics are shown in Table 1. Notably, the mean age was 157 days (SD 104), the mean day of illness at presentation was 4.13 (SD 1.71), and the most common virus was RSV, present in 52 subjects (75.4% of those tested).

**Table 1:** Demographics and Clinical Characteristics of infants presenting to the Pediatric Emergency Department with Acute Bronchiolitis (n = 82)

Demographics or Clinical Characteristic	n (%)
Sex	
Male	52 (63.4)
Female	30 (36.6)
Race	
White or Caucasian	46 (56.1)
Black or African American	25 (30.5)
Asian	1 (1.20)
Other	10 (12.2)
Ethnicity	
Hispanic	41 (50.0)
Non-Hispanic	41 (50.0)
Primary language	
English	68 (84.0)
Spanish	11 (13.6)
Other	2 (2.40)
Respiratory virus isolated	
Adenovirus	2 (2.90)
COVID-19	5 (7.25)
Human metapneumovirus	3 (4.35)
Parainfluenza Virus 3	6 (8.70)
Parainfluenza Virus 4	1 (1.43)
Rhinovirus/enterovirus	27 (39.1)
RSV	52 (75.4)
Disposition	
Discharge	22 (27.2)
Floor	40 (49.3)
ICU	19 (23.5)
Discharged and returned to the ED within 7 days and admitted?	
Yes	2 (9.10)
No	20 (90.9)
Escalated to the ICU if admitted to the floor?	
Yes	3 (7.50)
No	37 (92.5)
Admission diagnosis	
Bronchiolitis only	77 (93.9)
Bronchiolitis + pneumonia	5 (6.10)
Discharge diagnosis	
Bronchiolitis only	72 (87.8)
Bronchiolitis + pneumonia	10 (12.2)
ESI	
1	4 (4.88)
2	58 (70.7)
3	18 (21.9)
4	2 (2.44)
5	0 (0.00)
Mean (SD)	
Age (days)	157 (104)
Weight (kg)	7.19 (2.34)
Gestational age at birth (weeks)	38.5 (1.52)
Day of illness on presentation	4.13 (1.71)
ESI	2.22 (0.57)
Hospital LOS (hours)	84.5 (62.9)
Scanning time (minutes)	7.65 (1.45)

The mean scanning time for each technique is shown in Table 2. The mean waterfall scanning time was 1.65 min (SD 0.55, range 1–3), compared to 7.65 min (SD 1.45, range 5–14) for the lawnmower technique.

**Table 2:** Waterfall and lawnmower mean scan time and LUS scores by category

	Waterfall mean scan time (SD)	Lawnmower mean scan time (SD)
	1.65 min (SD 0.55)	7.65 min (SD 1.45)
	Waterfall mean LUS score (SD)	Lawnmower mean LUS score (SD)
By disposition		
Discharge	0.36 (0.66)	1.18 (1.33)
Floor	1.34 (1.32)	4.34 (3.62)
ICU	2.79 (1.81)	10.84 (6.54)
By RS* at time intervals†		
12 h		
No RS	0.44 (0.75)	1.56 (1.93)
Low RS	1.68 (0.57)	4.34 (3.51)
High RS	3.06 (1.85)	11.94 (6.17)
24 h		
No RS	0.56 (0.97)	2.11 (2.35)
Low RS	1.63 (1.31)	4.91 (3.86)
High RS	3.14 (1.79)	12.64 (6.48)
Maximum RS		
No RS	0.43 (0.73)	1.22 (1.31)
Low RS	1.19 (1.17)	4.11 (3.61)
High RS	2.82 (1.82)	10.45 (6.16)

^*^Respiratory support (RS) categories are as follows: no RS (room air), low RS (wall O2 and heated high flow nasal cannula < 1L/kg), and high RS (heated high flow nasal cannula ≥ 1L/kg, non-invasive positive pressure, e.g. BiPAP, and invasive ventilation)

^†^Times from when disposition decision order placed in emergency department. Maximum refers to entire hospitalization

Mean lawnmower total LUS score associations with RS at 12 and 24 h, maximum RS, and disposition were described previously (Table 2) [12]. Mean waterfall total LUS scores are described as follows and also reported in Table 2. At 12 h: no RS, 0.44 (SD 0.75); low RS, 1.68 (SD 0.57); and high RS, 3.06 (SD 1.85). At 24 h: no RS, 0.56 (SD 0.97); low RS, 1.63 (SD 1.31); and high RS, 3.14 (SD 1.79). For maximum RS during hospitalization: no RS, 0.43 (SD 0.73); low RS, 1.19 (SD 1.17); and high RS, 2.82 (SD 1.82).

We also previously reported mean lawnmower LUS scores based on disposition (Table 2) [12]. For the waterfall technique, mean total LUS scores are also seen in Table 2. The mean score for discharge from the ED was 0.36 (SD 0.66), for admission to the floor was 1.34 (SD 1.32), and for admission to the ICU was 2.79 (SD 1.81).

Comparisons of mean LUS scores at no, low, and high RS at 12 and 24 h, maximum RS, and disposition using the lawnmower technique were previously described. In brief, all differences in LUS scores were statistically significant (Table 3) [12]. Comparisons of the same using the waterfall technique were all statistically significant, except at maximum RS when comparing no RS to low RS (Table 3).

**Table 3:** Difference between mean LUS score comparisons

Disposition comparison	Difference between mean Waterfall LUS scores (CI)*	Difference between mean Lawnmower LUS scores (CI)*
Discharge-Floor	0.98 (0.06–1.57)	3.16 (0.56–5.75)
Floor-ICU	1.45 (CI 0.57–2.32)	6.50 (3.78–9.22)
Discharge-ICU	2.43 (CI 1.43–3.41)	9.66 (6.59–12.73)
RS comparison		
12 h		
No RS-Low RS	0.92 (CI 0.16–1.68)	2.79 (0.48–5.09)
Low RS-High RS	1.69 (CI 0.81–2.57)	6.60 (4.93–10.27)
No-High RS	2.61 (CI 1.68–3.55)	10.39 (7.55–13.22)
24 h		
No RS-Low RS	1.07 (CI 0.33–1.81)	2.80 (0.53–5.06)
Low RS-High RS	1.52 (CI 0.54–2.49)	7.74 (4.75–10.72)
No-High RS	2.59 (CI 1.63–3.54	10.53 (7.60–13.46)
Maximum RS		
No RS-Low RS	0.75 (CI -0.06–1.57)^	2.89 (0.31–5.47)
Low RS-High RS	1.63 (CI 0.80–2.46)	6.35 (3.73–8.69)
No-High RS	2.38 (CI 1.47–3.30)	9.24 (6.34–12.13)

^*^All comparisons statistically significant except as indicated by ^

To determine if a subject’s developmental age, and thus likelihood of being supine or upright, was significant, all mean waterfall LUS score comparisons were compared by age of ≤ 180 and > 180 days, or six months. As shown in Table 4, there was no statistically significant difference except for high RS at 24 h.

**Table 4:** Comparisons by age of waterfall mean LUS score and outcomes of interest (≤ 180 days vs > 180 days)

Disposition	Comparison between mean LUS scores (CI) of ≤ 180 days vs > 180 days
Discharge	0.24 (− 0.36–0.84)
Floor	0.54 (− 0.32–1.39)
ICU	1.48 (− 0.24–3.19)
RS	
12 h	
No RS	0.39 (− 0.22–0.996)
Low RS	0.24 (− 0.59–1.07)
High RS	1.71 (− 0.42–3.85)
24 h	
No RS	0.38 (− 0.28–1.04)
Low RS	0.02 (− 0.97–0.998)
High RS	2.30 (0.09–4.52)*
Maximum RS	
No RS	0.35 (− 0.29–0.99)
Low RS	0.35 (− 0.44–1.14)
High RS	0.80 (− 1.14–2.74)

^*^Indicates statistical significance

**Fig. 3:**
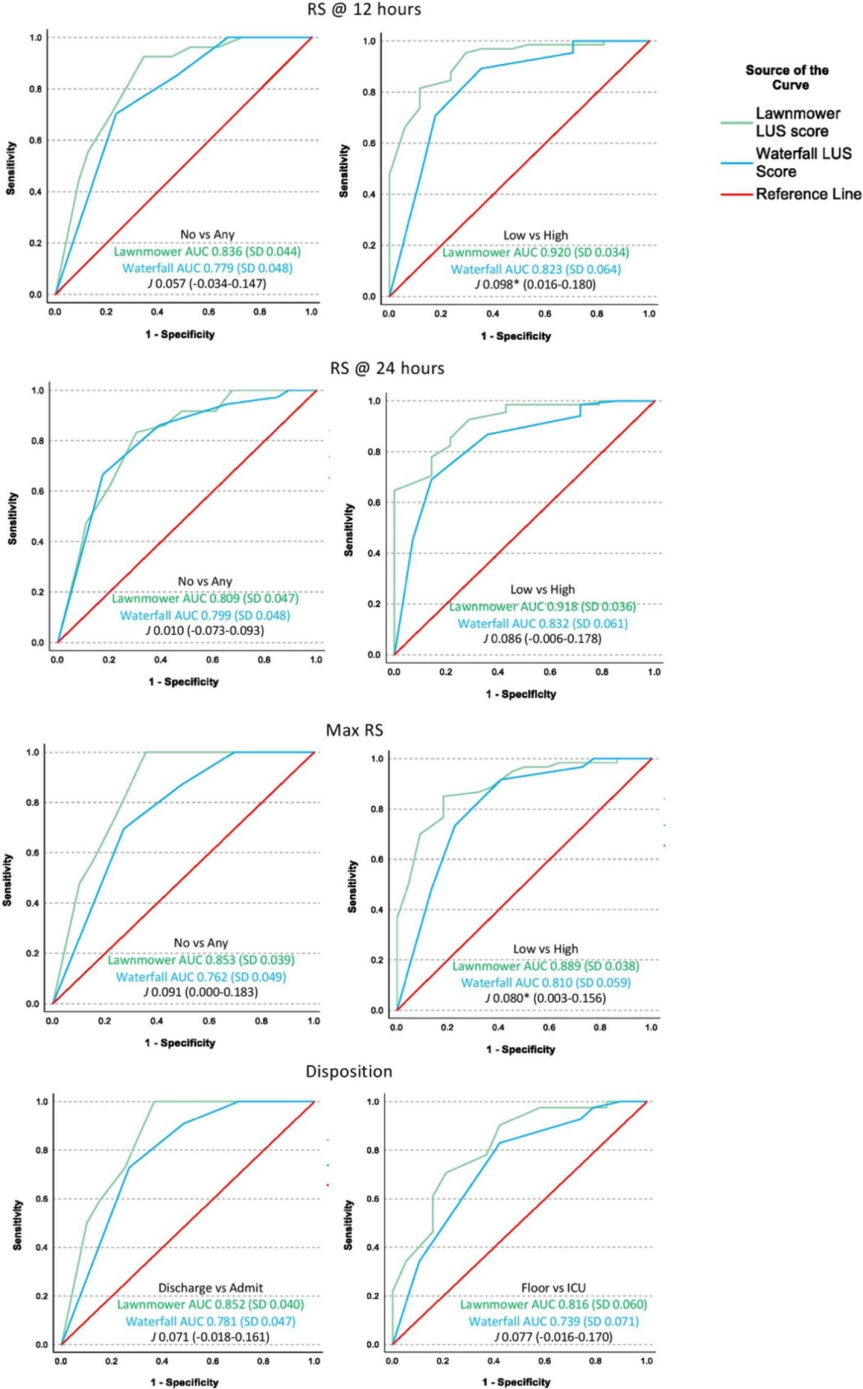
Receiver operating characteristic (ROC) curves comparing the lawnmower and waterfall LUS scores and RS at three time points: 12 h (1a), 24 h (2a), and maximum RS during hospitalization (1c), and three dispositions: discharge, floor, and ICU (1d). Since there were three RS categories, there are two ROC curves for each RS time point, no RS vs. any RS, and low RS vs. high RS. No RS is room air, low RS is wall O2 or HHFNC < 1L/kg, and high RS includes HHFNC ≥ 1L/kg, non-invasive and invasive positive pressure. Since there were three disposition categories, there are two ROC curves: discharge vs admit, and floor vs ICU. The area under the curve (AUC) Youden Index (J) is reported, with * indicating statistical significant

Comparing waterfall and lawnmower LUS scores for RS at 12, 24 h, maximum support, and disposition using ROC curves are seen in Fig. 3. AUC and Youden Index (*J*) for each are included. The difference between the AUCs (*J*) was statistically significant for the following comparisons: RS at 12 h comparing low RS and high RS (*J* 0.098, CI 0.016–0.180), and maximum RS comparing low and high RS (*J* 0.080, CI 0.003–0.156). There were no statistically significant differences in AUCs for any other comparisons between the waterfall LUS scores and lawnmower LUS scores.

The mean LOS was 84.5 h (SD 62.9). The Pearson correlation coefficient (r) for LOS and the waterfall LUS score was 0.646 (*p* < 0.00001) indicating a high degree of correlation. For the lawnmower technique, the (r) was 0.489 (*p* < 0.00001), which is moderate degree of correlation.

The IRR using Cohen’s weighted kappa (k) for the waterfall technique for retrieved scans was 0.824 (CI 0.733–0.915), indicating excellent agreement. The k for the 14 (17.1%) of subjects for which a second scan was performed was 0.503 (CI 0.253–0.752) indicating moderate agreement. This is in comparison to the respective k for the lawnmower technique of 0.935 (CI 0.899–0.971) and 0.662 (0.522–0.803).

## Discussion

LUS is an emerging diagnostic tool for several lung pathologies, including AB. Because of the paucity of reliable objective assessment tools for AB, bedside LUS appears primed to serve as a valuable, novel tool. However, because of its relative novelty, and the need to balance diagnostic accuracy and time for image acquisition, beside LUS is subject to variable techniques. Each scanning technique has differing accuracy, predictive value, and time considerations. Copetti et al., first described pediatric LUS in the literature in 2008 [28]. Since then, various techniques have been suggested, including different probe positioning, fields scanned, and time spent performing the scan. Liu et al. published protocols in 2019 consisting of scanning using a 6 or 12-lung segment view, with each area scanned in longitudinal and transverse orientations, as well as obtaining a transdiaphragmatic view [22]. This is inconsistently used [7, 11–15, 19–21, 29, 31, 33, 34]. Indeed, the time required to perform LUS as suggested can be impractical for some ED clinicians. Thus, identifying the technique that requires the least amount of time while retaining clinical predictive value is important to the ED clinician.

In our cohort of 82 pediatric subjects with AB, a posterior paraspinal scanning technique in longitudinal position, or waterfall approach, was markedly shorter to perform than our [12] and others’ previously published lawnmower technique [22, 28]. Additionally, the associations between the waterfall LUS score and RS at 12 h, 24 h, maximum support during hospitalization, disposition, and hospital LOS were nearly all statistically significant when analyzed independently. When both waterfall and lawnmower techniques were directly compared, all but two outcome comparisons were not statistically significantly different. Our data suggest that while the waterfall technique is somewhat less predictive of RS, it is comparable to the lawnmower technique in predicting disposition, and it has a strong association with hospital LOS.

Basile et al., and others have suggested that the posterior lung fields are most reflective of lung disease in AB and the strongest association with patient outcomes [7, 13, 27]. However, many previous LUS studies’ age cutoff was 6 months, that is, those who are developmentally more likely to be supine [13, 27]. Our study enrolled those up to 12 months to include subjects who are more frequently upright. We did not include subjects older than 12 months to decrease the likelihood of capturing AB-mimickers such as reactive airway disease and asthma. We performed a separate analysis that divided our data at the cutoff of 180 days, or six months, when infants are, on average, beginning to sit up [35]. We then compared the LUS scores with the endpoints of interest aforementioned. All but one comparison was not statistically significantly different, indicating that developmental age and likelihood of being supine does not appear to change the relevance of these previous studies. It should also be noted that Loi et al. [36], found that in neonatal subjects, patient positioning for certain durations changed the LUS score. This was not accounted for in our study, given that our patient population included older infants at different developmental stages, which would have made their positioning at a specific point in time difficult to capture.

The IRR kappa (k) for the waterfall technique, while excellent in agreement for image interpretation, was moderate for image acquisition. There are a few possible explanations for this difference. First, the IRR for image acquisition was only performed in 17.1% of subjects due to secondary scanner availability. Next, our waterfall scanning technique protocol required that the scanner perform the LUS in between the scapula and spine, but no measurements for this anatomical location were required or documented. Finally, given the patchy nature of bronchiolitis, it is possible that even a few millimeters difference could yield different results. While a transverse, rather than sagittal, orientation approach could have perhaps decreased some of this variability, the sagittal orientation was still employed to ensure an appropriate comparison with the lawnmower technique, which is also a sagittal orientation. We also used the sagittal plane to guarantee timeliness of the scan as this orientation allows for multiple lung spaces to be observed simultaneously.

The waterfall and lawnmower techniques used identical scoring criteria, but inherently had different potential total scores. As mentioned previously, we compared these scores using the AUC of ROC curves. However, with three categories of RS and three categories of disposition, this comparison initially proved difficult. We opted to compare the RS and the disposition in a way that was clinically meaningful. For example, we compared no RS to any RS, as well as low RS to high RS, but did not do a comparison of no RS to high RS as this is less useful to distinguish clinically. Similarly, we compared discharge and admission, as well as compared floor admission to ICU admission, but did not compare discharge to ICU admission as it is less clinically helpful.

## Limitations

There are limitations to consider in addition to those presented previously [12]. This study was conducted at a single, tertiary-care, pediatric-specific institution with ultrasound resources and training that may not be available at other institutions. As mentioned, the time required to scan for each technique was not exact, rather rounded to the nearest minute, although it is unlikely to have changed our results. Also, the time reported was obtained from the machine, based on actual scanning time. The time did not account for retrieving the machine, entering in patient information, and patient positioning. However, it is assumed that this would all be the same for each technique. The inherent nature of the waterfall technique and total potential score allow for a smaller margin of error compared to the lawnmower technique and may be less reproducible. As mentioned, exact measurements were not required of where the probe was to be placed other than in between the spine and scapula and in theory would be subject to variability and even bias since the waterfall scan was done after the lawnmower scan. However, this paraspinal space is quite small in infants and is likely negligible. Finally, in our analysis to differentiate if developmental stage of being supine influences posterior lung field score, we used the cutoff of 180 days, or 6 months, as a generic understanding of development, rather than based on patient-specific developmental status.

## Conclusion

Our study shows that, in infants diagnosed with AB in the pediatric ED, the waterfall technique for bedside LUS is faster than the lawnmower technique, comparable in association with disposition, and strongly associated with LOS. It is not, however, as strongly associated with RS. Prospective studies with larger populations across multiple sites are needed to confirm these findings and to compare additional LUS techniques not addressed in this study.

## Supplementary Information

Supplementary material 1. Scoring form filled out for physician performing lung ultrasound. For each of the 12 lung segments in the lawnmower technique, a score of 0-3 is indicated for a possible total score of 36. For the waterfall technique, there are two lung fields for a total possible score of 6

Supplementary material 2. The respiratory support (RS) by time intervals 12 and 24 hours, as well as maximum respiratory support for hospitalization. These respiratory supports were the original subdivisions, which were room air (RA), wall O2, heated high flow nasal cannula (HHFNC) <1L/kg, 1-2L/kg, and >2L/kg, and positive pressure which included noninvasive (ie CPAP and BiPAP) and invasive methods (intubation). The mean lung ultrasound score (LUS) was calculated for each RS and differences between the means were also calculated

## Data Availability

The datasets generated and analyzed during this study are not publicly available as they contain personal health information, but are available from the corresponding author on reasonable request.

## Abbreviations

AB: Acute bronchiolitis
LUS: Lung ultrasound
ED: Emergency department
RS: Respiratory support
AUC: Area under the curve
*(J)*: Youden index
IRR: Inter-rater reliability
LOS: Length of stay
SD: Standard deviation
CI: Confidence interval
ICU: Intensive care unit
ROC: Receiver operating characteristic
(k): Kappa
HHFNC: Heated high-flow nasal cannula
