# Supplementary material for: Comparison of two point-of-care lung ultrasound techniques and their associated outcomes for bronchiolitis in the pediatric emergency department
Source: Ultrasound J. 2025 Jan 17;17:8. doi: 10.1186/s13089-025-00410-y (PMC11748713; doi:10.1186/s13089-025-00410-y)
Supplement: Supplementary file 1 — Supplementary material 1. Scoring form filled out for physician performing lung ultrasound. For each of the 12 lung segments in the lawnmower technique, a score of 0-3 is indicated for a possible total score of 36. For the waterfall technique, there are two lung fields for a total possible score of 6 [file 13089_2025_410_MOESM1_ESM.docx]

Place patient label here

Supplement 1: Lung Ultrasound findings

Please mark the appropriate findings for each of the following lung fields, and include the score.

|  | Normal lung sliding, mostly A lines, and/or <3 B lines per lung segment (0 points) | ≥3 B lines per lung segment, but not consolidated or “white out” (1 point) | Consolidated B lines/"white out"  (2 points) | Any of the previous findings *and* presence of subpleural consolidations (include size cm)  (3 points) | Score (0-3) |
| --- | --- | --- | --- | --- | --- |
| L posterior |  |  |  |  |  |
| R posterior |  |  |  |  |  |

Start time of waterfall examination:________________ End time of waterfall examination:______________

Total Score (0-6):_______________

Start time of 12-field examination:______________ End time of 12-field examination:_____________

|  | Normal lung sliding, mostly A lines, and/or <3 B lines per lung segment (0 points) | ≥3 B lines per lung segment, but not consolidated or “white out” (1 point) | Consolidated B lines/"white out"  (2 points) | Any of the previous findings *and* presence of subpleural consolidations (include size cm)  (3 points) | Score (0-3) |
| --- | --- | --- | --- | --- | --- |
| L anterior superior |  |  |  |  |  |
| L anterior inferior |  |  |  |  |  |
| L lateral superior |  |  |  |  |  |
| L lateral inferior |  |  |  |  |  |
| L posterior superior |  |  |  |  |  |
| L posterior inferior |  |  |  |  |  |
| R anterior superior |  |  |  |  |  |
| R anterior inferior |  |  |  |  |  |
| R lateral superior |  |  |  |  |  |
| R lateral inferior |  |  |  |  |  |
| R posterior superior |  |  |  |  |  |
| R posterior inferior |  |  |  |  |  |

Total Score (0-36):_______________

Scoring form filled out for physician performing lung ultrasound. For each of the 12 lung segments in the lawnmower technique, a score of 0-3 is indicated for a possible total score of 36. For the waterfall technique, there are two lung fields for a total possible score of 6.
