# Supplementary material for: Comparison of two point-of-care lung ultrasound techniques and their associated outcomes for bronchiolitis in the pediatric emergency department
Source: Ultrasound J. 2025 Jan 17;17:8. doi: 10.1186/s13089-025-00410-y (PMC11748713; doi:10.1186/s13089-025-00410-y)
Supplement: Supplementary file 2 — Supplementary material 2. The respiratory support (RS) by time intervals 12 and 24 hours, as well as maximum respiratory support for hospitalization. These respiratory supports were the original subdivisions, which were room air (RA), wall O2, heated high flow nasal cannula (HHFNC) <1L/kg, 1-2L/kg, and >2L/kg, and positive pressure which included noninvasive (ie CPAP and BiPAP) and invasive methods (intubation). The mean lung ultrasound score (LUS) was calculated for each RS and differences between the means were also calculated [file 13089_2025_410_MOESM2_ESM.docx]

| **RS by time interval** | **Mean LUS score (SD)** |  |
| --- | --- | --- |
| **12 hours** |  |  |
| RA | 0.44 (0.75) |  |
| Wall O2 | 1.60 (1.65) |  |
| HHFNC <1L/kg | 1.29 (1.08) |  |
| HHFNC 1-2L/kg | 2.20 (1.55) |  |
| HHFNC >2L/kg | 4.33 (1.15) |  |
| Positive Pressure | 4.25 (2.06) |  |
| **24 hours** |  |  |
| RA | 0.56 (0.97) |  |
| Wall O2 | 2.17 (1.17) |  |
| HHFNC <1L/kg | 1.50 (1.33) |  |
| HHFNC 1-2L/kg | 3.25 (1.58) |  |
| HHFNC >2L/kg | 5 (0) |  |
| Positive Pressure | 2.60 (2.19) |  |
| **Maximum** |  |  |
| RA | 0.43 (0.73) |  |
| Wall O2 | 1.18 (1.72) |  |
| HHFNC <1L/kg | 1.19 (0.90) |  |
| HHFNC 1-2L/kg | 2.25 (1.54) |  |
| HHFNC >2L/kg | 4.33 (1.15) |  |
| Positive Pressure | 3.14 (2.19) |  |
|  |  |  |
| **RS by time interval** | **Difference between mean LUS scores (CI)** | ***p* value <0.05? (Y/N)** |
| **12 hours** |  |  |
| RA-wall O2 | 1.16 (-0.13-2.44) | N |
| RA-HHFNC <1L/kg | 0.84 (-0.10-1.78) | N |
| RA-HHFNC 1-2L/kg | 1.76 (0.47-3.04) | Y |
| RA-HHFNC >2L/kg | 3.89 (1.77-6.01) | Y |
| RA-positive pressure | 3.81 (1.94-5.67) | Y |
| Wall O2-HHFNC <1L/kg | -0.31 (-1.60-0.9685) | N |
| Wall O2-HHFNC 1-2L/kg | 0.60 (-0.96-2.16) | N |
| Wall O2-HHFNC >2L/kg | 2.73 (0.44-5.03) | Y |
| Wall O2-positive pressure | 2.65 (0.59-4.51) | Y |
| HHFNC <1L/kg-HHFNC 1- 2L/kg | 0.91 (-0.37-2.20) | N |
| HHFNC<1L/kg-HHFNC >2L/kg | 3.05 (0.93-5.16) | Y |
| HHFNC <1L/kg-positive pressure | 2.96 (1.10-4.83) | Y |
| HHFNC 1-2L/kg-HHFNC >2L/kg | 2.13 (-0.16-4.43) | N |
| HHFNC 1-2L/kg-positive pressure | 2.05 (-0.01-4.11) | N |
| HHFNC >2L/kg-positive pressure | -0.0833 (-2.74-2.58) | N |
| **24 hours** |  |  |
| RA-wall O2 | 1.61 (-0.02-3.24) | N |
| RA-HHFNC <1L/kg | 0.94 (-0.005-1.89) | N |
| RA-HHFNC 1-2L/kg | 2.69 (1.25-4.14) | Y |
| RA-HHFNC >2L/kg | 4.44 (0.28-3.80) | Y |
| RA-positive pressure | 2.04 (6.36-17.02) | Y |
| Wall O2-HHFNC <1L/kg | -0.67 (-2.34-1.00) | N |
| Wall O2-HHFNC 1-2L/kg | 1.08 (-0.91-3.08) | N |
| Wall O2-HHFNC >2L/kg | 2.83 (-1.15-6.82) | N |
| Wall O2-positive pressure | 0.43 (-1.80-2.67) | N |
| HHFNC <1L/kg-HHFNC 1-2L/kg | 1.75 (0.26-3.24) | Y |
| HHFNC<1L/kg-HHFNC >2L/kg | 3.50 (-0.26-7.26) | N |
| HHFNC <1L/kg-positive pressure | 1.10 (-0.70-2.90) | N |
| HHFNC 1-2L/kg-HHFNC >2L/kg | 1.75 (-2.16-5.66) | N |
| HHFNC 1-2L/kg-positive pressure | -0.65 (-2.75-1.45) | N |
| HHFNC >2L/kg-positive pressure | -2.40 (-6.44-1.64) | N |
| **Maximum RS** |  |  |
| RA-wall O2 | 0.75 (-0.59-2.09) | N |
| RA-HHFNC <1L/kg | 1.82 (0.51-3.12) | Y |
| RA-HHFNC 1-2L/kg | 1.82 (0.51-3.12) | Y |
| RA-HHFNC >2L/kg | 3.90 (1.65-6.15) | Y |
| RA-positive pressure | 2.71 (1.13-4.29) | Y |
| Wall O2-HHFNC <1L/kg | 0.01 (-1.31-1.33) | N |
| Wall O2-HHFNC 1-2L/kg | 1.07 (-0.46-2.60) | N |
| Wall O2-HHFNC >2L/kg | 3.15 (0.77-5.54) | Y |
| Wall O2-positive pressure | 1.96 (0.19-3.73) | Y |
| HHFNC <1L/kg-HHFNC 1-2L/kg | 1.06 (-0.22-2.34) | N |
| HHFNC<1L/kg-HHFNC >2L/kg | 3.14 (0.91-5.37) | Y |
| HHFNC <1L/kg-positive pressure | 1.95 (0.39-3.51) | Y |
| HHFNC 1-2L/kg-HHFNC >2L/kg | 2.08 (-0.28-4.45) | N |
| HHFNC 1-2L/kg-positive pressure | 0.89 (-0.85-2.63) | N |
| HHFNC >2L/kg-positive pressure | -1.19 (-3.72-1.34) | N |

Abbreviations

RS – respiratory support

LUS – lung ultrasound

SD – standard deviation

CI – confidence interval

RA – room air

HHFNC – heated high flow nasal cannula

The respiratory support (RS) by time intervals 12 and 24 hours, as well as maximum respiratory support for hospitalization. These respiratory supports were the original subdivisions, which were room air (RA), wall O2, heated high flow nasal cannula (HHFNC) <1L/kg, 1-2L/kg, and >2L/kg, and positive pressure which included noninvasive (ie CPAP and BiPAP) and invasive methods (intubation). The mean lung ultrasound score (LUS) was calculated for each RS and differences between the means were also calculated.
